# In Silico Optimization of Femoral Fixator Position and Configuration by Parametric CAD Model

**DOI:** 10.3390/ma12142326

**Published:** 2019-07-22

**Authors:** Nikola Korunovic, Dragan Marinkovic, Miroslav Trajanovic, Manfred Zehn, Milorad Mitkovic, Saverio Affatato

**Affiliations:** 1 Faculty of Mechanical Engineering, University of Nis, 18000 Nis, Serbia; 2 Dept of Mechanics, Technical University of Berlin, 10623 Berlin, Germany; 3 Faculty of Medicine, University of Nis, 18000 Nis, Serbia; 4 Serbian Academy of Sciences and Arts, Belgrade, 11000 Belgrade, Serbia; 5 Laboratorio di Tecnologia Medica, IRCCS - Istituto Ortopedico Rizzoli, 40136 Bologna, Italy

**Keywords:** orthopedic, fixator, parametric CAD model, finite element method (FEM), structural optimization

## Abstract

Structural analysis, based on the finite element method, and structural optimization, can help surgery planning or decrease the probability of fixator failure during bone healing. Structural optimization implies the creation of many finite element model instances, usually built using a computer-aided design (CAD) model of the bone-fixator assembly. The three most important features of such CAD models are: parameterization, robustness and bidirectional associativity with finite elements (FE) models. Their significance increases with the increase in the complexity of the modeled fixator. The aim of this study was to define an automated procedure for the configuration and placement of fixators used in the treatment of long bone fractures. Automated and robust positioning of the selfdynamisable internal fixator on the femur was achieved and sensitivity analysis of fixator stress on the change of major design parameters was performed. The application of the proposed methodology is considered to be beneficial in the preparation of CAD models for automated structural optimization procedures used in long bone fixation.

## 1. Introduction

Internal fixation based on biological, rather than mechanical priorities (biological internal fixation) represents a well-established approach for treating long bone fractures, especially in the proximal femur [1]. It allows, and even requires, that fractured segments remain mutually mobile [2]. While such mobility is often beneficial for the formation of a callus, it results in substantial loading of the applied fixation device, which may cause stability, strength, or durability related issues [3,4,5]. To prevent these problems, structural analysis and structural optimization [6] are often used. Today, structural analysis of bone-fixator systems is, almost exclusively, performed using the finite element method (FEM). For a known fixator configuration and position relative to the bone, structural analysis is employed to assess bone and fixator deformations, stresses, and strains, which are related to the fixator durability and the success of bone healing. Structural analysis has often been used to compare the suitability of various fixation devices for a certain fracture type, e.g. [7,8,9,10,11,12]. Parametric studies or optimization procedures have been employed to find the optimal configuration and position of an existing fixation device [13,14,15,16,17] or to optimize the shape and dimensions of a new one (e.g. [18]).

The geometry of finite element (FE) models of bones and fixators is typically based on underlying computer-aided design (CAD) models. With regard to CAD and FE models of bones, the most important concerns are representation of complex bone geometry [19,20,21,22,23,24,25,26], characterization of specific material properties [23,24,25], and universal definition of anatomical landmarks [24,25,27]. Anatomical landmarks are used as positioning markers in the creation of the bone-fixator assembly. Fixator models are simpler to build than bone models, as their shapes and material properties are less complex. However, care must be taken to create suitable references for their assembly with bone models. Finally, the appropriate modeling of boundary conditions and loads must be applied in order for FE analysis results to be valid [28,29,30].

Similar to other fixation devices, the selfdynamisable internal fixator (SIF) represents the ultimate standard in internal fixation of long bones and in the healing of fractures without mechanical failure (e.g., bending of the bar or breaking of screws) or any other complication during the healing process. SIF is a medical device characterized by a modular structure, extensively used by Mitkovic et al. [31,32]. In their studies, these authors applied this medical device on 726 patients (in the healing of proximal, diaphyseal, and distal femur fractures). They observed that screw breaking occurred in 2.6% of the fixations and that the bar broke at the connection to the trochanteric unit in 0.3% cases [31]. The recorded percentage of SIF failures was considered to be small, but it also showed that there is room for improvement in SIF durability. 

The application of SIF in the fixation of common fractures, if performed by experienced orthopedic surgeons, represents a routine process. Nevertheless, if a complex fracture is treated and/or surgery is approached by an orthopedic surgeon lacking sufficient experience, the choice of the appropriate SIF components and its placement on the bone can be more complex. Compared to other devices used in the fixation of long bones, its complexity is also greater. It is characterized by a modular design similar to the design of external fixators [31] and contains additional clamps and more screws than most of the other fixation devices [3]. Consequently, the parameterization of the SIF’s position on the femur, in relation to CAD modeling, represents a more challenging task. For this reason, the methodology for SIF positioning in relation to the underlying femur was developed to ensure the robustness of the femur–SIF assembly versus any possible change in SIF configuration.

The main aim of our research was to define an automated procedure for the optimization of configuration and placement of fixators used in the treatment of long bone fractures. More specifically, our goal was to prove that SIF positioning in relation to the underlying femur, within the CAD model of the femur–SIF assembly, could be parameterized in such a way that all possible changes in SIF configuration resulted in a valid CAD model of the femur–SIF assembly.

## 2. Materials and Methods 

### 2.1. Fixation Device

**Figure 1:**
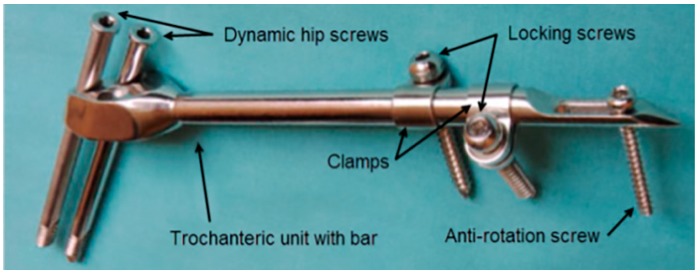
Components of a selfdynamisable internal fixator (SIF) configured for subtrochanteric femoral fracture treatment.

A typical SIF configuration is shown in Figure 1. Two dynamic hip screws and two of the three dynamic hip screw holes in the trochanteric unit are used. The top hole is always used to place the screw directed towards the center of the femoral head and the choice of the other hole depends on whether the right or the left femur is fixed. The clamps may freely rotate around the bar and each of the clamps may be positioned to have the locking screw hole on either side of the bar. By doing so, an excellent 3D stability of the fixator can be achieved [31]. The anti-rotation screw limits any axial movement and contributes to the rotational stability of the fixator.

### 2.2. Approach to CAD Modeling of the Femur–SIF Assembly

In this study, a non-parametric subject-specific solid CAD model of the human femur obtained from a CT scan, described in [33], was used to represent bone geometry. Materialise Mimics (ver. 17 Materialise, Leuven, Belgium) and Dassault Systèmes CATIA (V5R21, Dassault Systèmes, Paris, France) were used to create the surface model of the bone from medical images. The bone was modeled as a homogenous solid. This approximation was introduced in order to simplify the model and focus on the parameterization of SIF configuration and placement. The CAD model of the bone was prepared for assembly with the parametric CAD model of the fixator, by creating the necessary landmarks, namely anatomical points, axes, curves, and planes. All those objects were created on the femur model in order to serve as geometrical references for positioning the fixator and for the definition of loads and boundary conditions in subsequent finite element analysis (FEA). In the current study, this preparation was performed manually according to a predefined procedure, while the ultimate goal is to have the whole procedure automated. A fully parametric CAD model of SIF was used, and its position on the femur was defined via parametric constraints. SolidWorks (ver. 2015, Dassault Systèmes, Paris, France) was used to create the SIF model and the femur–SIF assembly. 

### 2.3. Anatomical Landmarks on the CAD Model of the Femur

**Figure 2:**
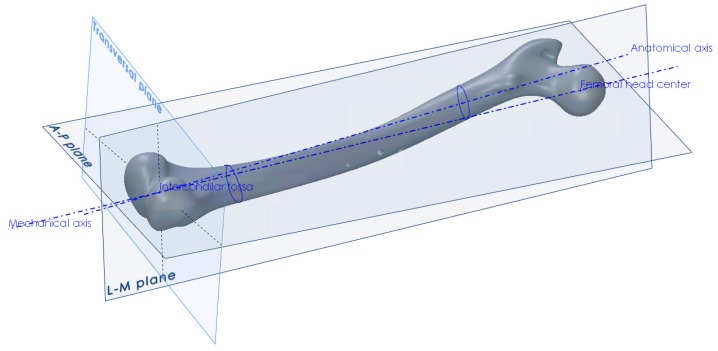
Creation of anatomical points, axes and planes on the femur.

The procedure for the creation of anatomical planes, required that further work, started with the determination of the point of the intercondylar fossa and the center point of the femoral head [34]. The direction between those two points defined the mechanical axis, which in turn defined the direction of the force vector that would represent the human weight in the finite element analysis (Figure 2). Another axis that needed to be created, in order to construct the anterior-posterior plane, was the anatomical axis. This was constructed by connecting the centers of gravity of the two femoral shaft cross-sections, one at its proximal and the other at its distal end. After obtaining the required axes, the A–P plane was constructed, which coincided with the mechanical and anatomical axes. The L–M plane was then constructed, coincident with the mechanical axis and perpendicular to the A–P plane (Figure 2). The creation of all necessary points, axes, curves and planes is described in detail in [34]. Besides the creation of landmark points and axes, the centers of gravity of several femoral cross-sections parallel to the horizontal plane were interpolated using a spline object, in order to create the anatomical curve.

### 2.4. SIF Configuration and Assembly Constraints

**Figure 3:**
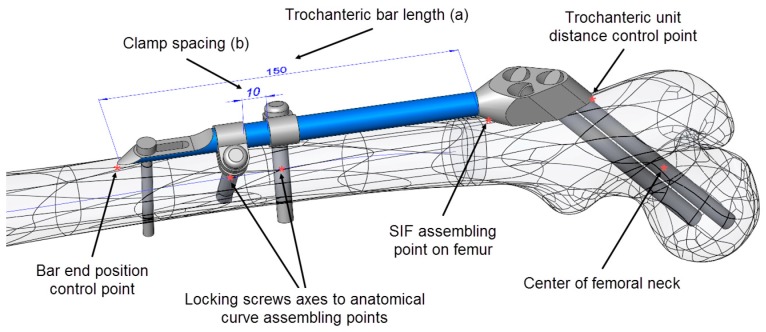
Driving design parameters (bar length (**a**) and clamp spacing (**b**)) and specific points on the computer-aided design (CAD) model.

For the positioning of the fixator on the femur, several specific points on the CAD model of the femur (Figure 3), as well as the anatomical curve, were used. The choice of reference points and assembly constraints was crucial for the robustness of the whole femur–SIF assembly, which implied that after an arbitrary change of main design parameters inside the allowed limits, the mutual position of the femur and SIF still satisfied the orthopedist’s requirements.

As SIF is modular and the change of module shapes was not considered, the choice of variable design parameters was restricted to assembly constraints, which an orthopedist could change during surgery. The following driving design parameters were defined in the assembly (Figure 3):

- Bar length (discrete)
- Clamp spacing (continuous)

To adjust SIF to a certain fracture type and location, the orthopedic surgeon can choose from 4 bar lengths: 100, 150, 200, and 250 mm. If two clamps are used, which is most often the case, their mutual position is generally arbitrary. Nevertheless, the distance between the clamps (clamp spacing) also dictates the length of the distal surgical cut created during operation (where less is better). In this case, an interval of 1 to 28 mm was considered representative.

During surgery, anatomical landmarks, such as approximate points on trochanters, femoral head or neck, are used to determine the initial position of the fixator on the femur, while fluoroscopy (live X-ray imaging) is used to find its exact position. Descriptive empirical constraints are thereby used by surgeons. For example, the surgeons take care that the first dynamic hip screw passes near the center of the femoral head, with its tip finishing five millimeters away from the femoral head surface and that the second dynamic hip screw stays a couple of millimeters away from the femur neck’s surface. One of the tasks of this research was to replace those empirical constraints with design parameters and constraints where, in some cases, the landmarks used by surgeons had to be replaced by different ones. This is illustrated next.

**Figure 4:**
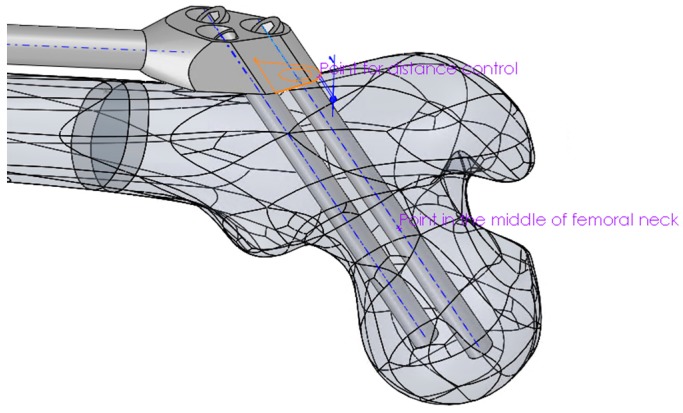
Positioning of the proximal part of the trochanteric unit relative to the femur.

Mimicking the logic of a surgeon, the proximal part of the trochanteric unit was at first positioned in such a way that the axis of the proximal dynamic hip screw passed through the central point of the femoral head and through an appropriate breakthrough point on the lateral femoral surface. The breakthrough point was adjusted so that both proximal and distal dynamic hip screws stayed inside the bone, a few millimeters away from the bone surface. Thus, in the first modeling attempt, the breakthrough point of the proximal dynamic hip screw was created as a specific point on the bone’s surface. This point was parametrically defined as a point on a corresponding surface patch of the CAD model so that its position could be arbitrarily adjusted. However, the obtained bone-SIF assembly was not fully robust. The position of the fixator was not always appropriate, and an adjustment of the breakthrough point position was necessary every time the bar length was changed. In order to avoid such an inconsistency, a fixed point was created in the center of the femoral neck, which was used as a pivoting point of the proximal dynamic screw axis. This feature, in combination with three additional constraints, enabled robust positioning of the trochanteric unit with the bar on the femur. Those additional constraints were:

1. The coincidence of the symmetry plane of the trochanteric unit and a newly introduced point on the intersection of the femur surface and the A–P plane (the “SIF assembling point on femur” in Figure 3), placed distally from the initial breakthrough point on the femur surface and proximally from the fracture, at equal distances.
2. The distance between the trochanteric unit and the femur surface (Figure 4).
3. The positioning of the bar end, such that it closely followed the anatomical axis. This was achieved by projecting the anatomic curve on the femur surface in the direction parallel to the symmetry plane of the fixator and by creating an assembly component containing a single point, which was assembled both to the projected curve and the fixator end.

### 2.5. FE Model and Simulation

**Figure 5:**
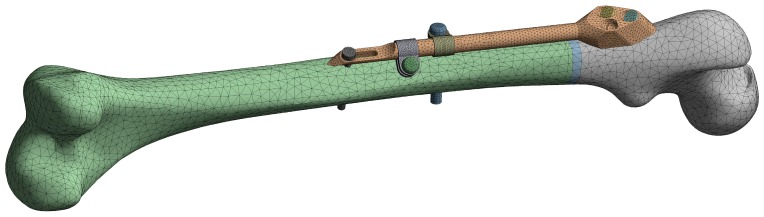
Finite element (FE) model of the femur–SIF assembly.

The initial FE model was based on the CAD model of the femur–SIF assembly with the default values of design parameters (a = 150 mm and b = 10 mm), imported into the ANSYS Workbench (ver. 17.1, Ansys Inc., Canonsburg, PA, USA) (Figure 5). Bidirectional associativity was thereby established, which enabled an automatic update of the FE model with a change of design parameters in the CAD model. 

The quadratic tetrahedron elements were used to create the mesh via the patch conforming algorithm (a Delaunay tetra mesher with an advancing-front point insertion technique used for mesh refinement [35]). The characteristic element size was set to 6 mm for the femur, 2 mm for the trochanteric unit with bar, dynamic hip screws and locking screw, and 1 mm for the anti-rotation screw, clamps and contact areas between the anti-rotation screw and the bar. The resulting mesh on the initial FE model was composed of 79122 elements. Bonded contact was defined between the fixator components and the femur, while frictional contact was defined between the fixator components, with the friction coefficient equal to 0.7. The linear material model was assigned to all assembly components, using the parameters given in Table 1. The elastic modulus of the bone, which was modeled as a single volume, was chosen to produce, under a given load, deflections similar to those of the real bone [36]. The elastic modulus of the callus was chosen to model callus stiffness three weeks after the surgery [14]. 

To test the behavior of the FE model, structural analysis was performed on various FE model instances (characterized by different values of design parameters), under the representative loading conditions similar to the one-legged stance (i.e., to the corresponding experiment that is often performed in laboratories) [37]. More precisely, the distal part of the femur was encastered (along surfaces of condyles and epicondyles, approximately up to the height of the intercondylar fossa), while a force of 883 N, corresponding to a patient’s mass of 90 kg, was imposed on the femoral head, acting in the direction of the anatomical axis. A geometrically nonlinear analysis was performed, which accounted for the large deflection effects. This analysis was adopted for the whole study, as the initial tests demonstrated that the difference in maximum values of the total deflection obtained by linear and geometrically nonlinear analyses exceeded 30%, and the difference in maximum values of the equivalent stress was greater than 20%. Another source of nonlinearity was the presence of frictional contact. Automatic loading incrementation was used, which typically resulted in 4 load increments (where the initial increment was equal to 20% of the load) and around 30 equilibrium iterations per analysis.

**Figure 6:**
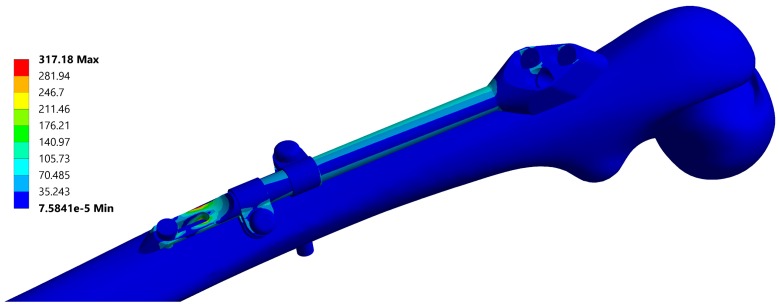
Equivalent stress field of the fixator, corresponding to the instance no. 6 (Table 2). Maximal stress values are typically located at the end of the bar.

**Figure 7:**
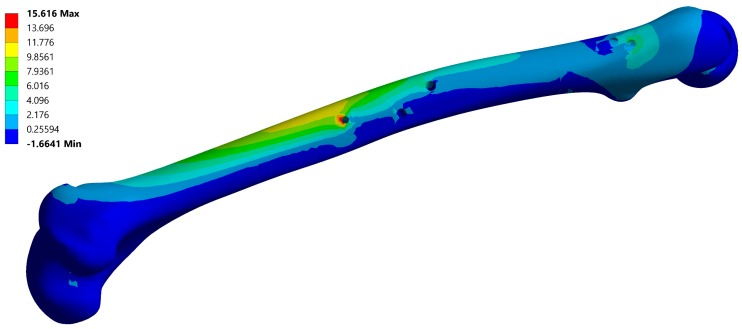
Maximum principal stress field of the femur, corresponding to instance no. 6 (Table 2). Maximal stress values are typically located on the anti-rotation screw hole.

A typical resulting stress field of the fixator is depicted in Figure 6 and a typical stress field of the femur is shown in Figure 7.

## 3. Results

### 3.1. Instances of Femur–SIF CAD and FE Models

**Figure 8:**
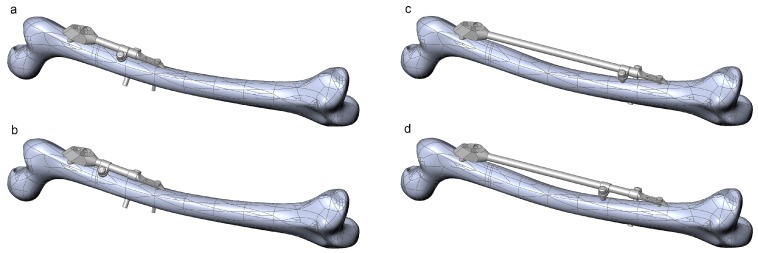
Instances of the femur–SIF assembly created using extreme combinations of design parameters. (**a**) Bar length 100 mm, clamp spacing 1 mm. (**b**) Bar length 100 mm, clamp spacing 28 mm. (**c**) Bar length 250 mm, clamp spacing 1 mm. (**d**) Bar length 250 mm, clamp spacing 28 mm.

The robustness of the CAD model of the femur–SIF assembly was checked through the creation of 16 CAD model instances (Table 2). All instances were rebuilt successfully, without the need for further user intervention or further adjustment of values of some of the parameters defining femoral landmarks. Instances with extreme combinations of design parameters are shown in Figure 8.

**Figure 9:**
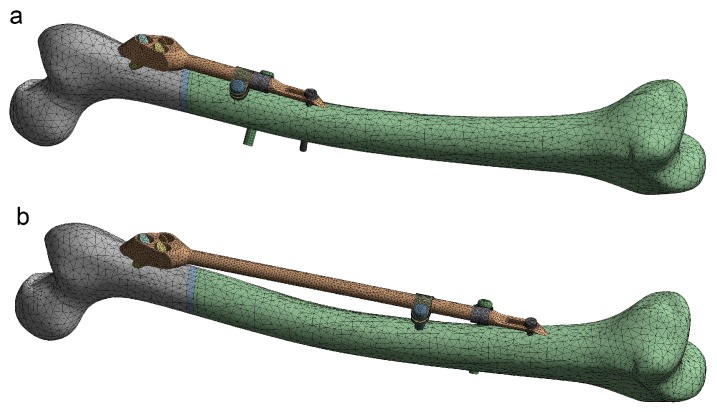
Instances of the femur–SIF FE model (only two instances are shown, as the others are similar). (**a**) Bar length 100 mm, clamp spacing 1 mm. (**b**) Bar length 250 mm, clamp spacing 28 mm.

The robustness of the FE model of the femur–SIF assembly was checked through the translation of all CAD model instances (Figure 9). All sixteen instances were imported into the ANSYS Workbench (ver. 17.1, Ansys Inc., Canonsburg, PA, USA) without any issues.

### 3.2. Sensitivity Study

**Figure 10:**
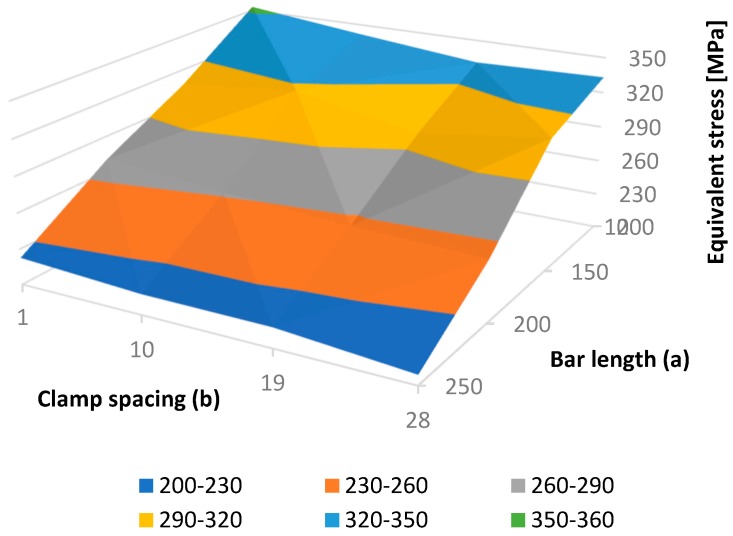
Equivalent fixator stress as a function of bar length (**a**) and clamp spacing (**b**).

Using the results of 16 finite element analyses, performed on all FE model instances, a dependence of the maximal fixator stress on bar length and clamp spacing was found, as presented in Table 2 and Figure 10. In all cases, the maximal fixator and bone stresses were significantly lower than the critical values. In general, the maximal fixator stress became lower with an increase in bar length and clamp spacing. The smoothness of the resulting surface (i.e., the exitance of a monotonic stress trend with the change of each parameter) indicates the quality of the FE model.

## 4. Discussion

In this study we explored a procedure for the optimization of configuration and placement of fixators used in the treatment of long bone fractures. The three main features of a CAD model designed for structural optimization are parameterization, robustness, and bidirectional associativity with an FE model. These were illustrated in the example of a dedicated CAD model of the femur–SIF assembly. 

SIF positioning, within the CAD model of the femur–SIF assembly, was successfully parameterized to enable the creation of a valid CAD model for every possible SIF configuration used in the fixation of subtrochanteric fractures. The choice of femoral landmarks and assembling references was, therefore, crucial. Alternative ways of referencing were found to enable the successful creation of all possible assembly configurations. For example, the reference axis on the femur, which served as a guide for the first dynamic screw, was created as a line passing through the center of the femoral neck and near the center of the femoral head. Thus, the screw breakthrough point, which is normally the point that the orthopedist chooses based on experience, was not created directly. Instead, it was defined as the intersection of the previously defined screw axis and femoral surface. Thus, the performance of automated optimization studies, based on CAD and FE models of SIF, was made possible. This was proved by performing a preliminary sensitivity study, in which the two main design parameters were changed at four levels each, to create, and sequentially solve, 16 different cases (design points) without a single problem related to CAD model robustness. The accent was also put on testing the parameterization and robustness of CAD and FE models. Thus, some physical properties, boundary conditions, and loads related to the FE model were simplified or taken from the literature.

To the authors’ knowledge, no reports evidenced that the position and configuration of a fixation device as complex as SIF had been parameterized before. Konya and Verim [13] performed the position optimization of proximal locking screws used in the proximal femoral nail system. In their study, two angles and one distance defining the position of the locking screw were defined as design parameters. Optimization studies of the femoral stem for total hip replacement are reported by Ishida et al. [38] and Chanda et al. [39], in which the geometry of various stem cross-sections was parameterized, but the position of the stem in relation to the femur was not. A study was reported by Chen et al. [40], in which the shape of a custom-designed fracture fixation plate was defined as a projection of the underlying bone, and only the width of the plate was parameterized. Another approach, by Bah et al. [41], used mesh morphing to change the position of the femoral stem in relation to the femur, which was possible because only micromotions were considered. 

The strengths of our study are mainly related to the fact that the underlying femur was characterized by an irregular shape (a highly curved shaft), and the variation of the SIF position, with the change of bar length, was rather extreme. Nevertheless, the positioning of SIF according to the developed methodology is neither absolute nor independent from a CAD designer, although care has been taken to minimize the influence of the designer’s actions on the result. Our study, obviously, has a number of limitations. First, it is related only to the application of SIF in the fixation of subtrochanteric fractures [32]. It may be assumed that a similar approach is valid for other SIF applications [31]. Second, the study was performed on a single subject-specific femur. Third, the only CAD program tested was SolidWorks, which was also chosen because of a two-way connection with FE models in ANSYS. In addition, our method was generated to be software independent, but some CAD programs may still not be able to recreate the CAD model geometry in an appropriate way.

Further studies should be performed in order to confirm the robustness of the CAD models in relation to the femur geometry and the designer’s actions related to landmark creation. The method should be tested on a number of femur geometries, including the “standardized femur” [42], and its robustness should be assessed using statistical analysis, e.g., Six Sigma tools or Taguchi methods.

Our results are considered to be significant as they enable the performance of sensitivity and optimization studies, which may help surgeons in a number of ways. First, these studies may reveal the general relations between deflections, strains, and stresses in the fixator and the underlying bone and the parameters that drive the design and position of the fixator. In this way, they may help orthopedists during the surgery planning process and reduce the probability of fixator failure during the healing process. Second, they may directly yield the optimal values of parameters for deflections, stresses, and fixator mass and make the orthopedist’s job even easier. 

The results of this study also bring the current research one step closer to the automatized optimization of the placement, configuration, and shape of SIF and similar fixation devices. Further studies will be directed to the creation of a fully automatic patient-specific procedure for the structural optimization of fixator configuration, i.e., a method that may regularly be used in orthopedics.

## 5. Conclusions

The positioning of the SIF on the femur, within the CAD model of the femur–SIF assembly, can be parameterized in such a way that the validity of the CAD model is retained for all possible changes in the SIF configuration. The FE model characterized by bidirectional associativity with the CAD model can be created successfully, reflecting all possible changes of design parameters. The CAD and FE models can be used in the automated optimization of the SIF configuration and position, which may serve as an aid in clinical practice. A similar approach can be taken for the creation of CAD models related to any fixation device similar to SIF, including external fixators.

## Figures and Tables

**Table 1:** Linear material model parameters of the femur–SIF assembly components.

Component	Material	Elastic Modulus (GPa)	Poisson’s Ratio	Yield Strength (MPa)
SIF	Stainless steel (ASTM F138-03)	210	0.3	min. 680
Femur	Bone	5	0.25	105
Fracture zone	Callus	1.16	0.25	105

**Table 2:** CAD model instances, the corresponding values of the design parameters, and the calculated maximal fixator stress.

Instance Number	Bar Length (a) (mm)	Clamp Spacing (b) (mm)	Maximal Fixator Stress (MPa)
1	100	1	353.26
2	100	10	341.41
3	100	19	330.54
4	100	28	333.15
5	150	1	307.04
6	150	10	317.18
7	150	19	297.94
8	150	28	312.13
9	200	1	270.38
10	200	10	261.84
11	200	19	255.28
12	200	28	251.45
13	250	1	222.59
14	250	10	217.08
15	250	19	216.63
16	250	28	208.15
